# Diagnosis and treatment of primary central nervous system lymphoma: A report of nine cases and literature review

**DOI:** 10.3892/ol.2015.2903

**Published:** 2015-01-27

**Authors:** JUN WANG, ZONGZE GUO, ERMENG MA, DEGUANG XING, BO QIU, YUNJIE WANG

**Affiliations:** Department of Neurosurgery, The First Hospital of China Medical University, Shenyang, Liaoning 110001, P.R. China

**Keywords:** lymphoma, stereotactic biopsy, radiotherapy, chemotherapy

## Abstract

Primary central nervous system lymphoma (PCNSL) is a rare, highly malignant tumor type of the nervous system and is associated with poor prognosis. To investigate the efficacy of current treatment strategies for PCNSL, the present study retrospectively analyzed the clinical and pathological features, imaging results, clinical management, and prognoses of nine patients with PCNSL. Postoperative pathological examination confirmed a diagnosis of lymphoma in all the patients and the adopted treatment regimens were as follows: Stereotactic biopsy in combination with methylprednisolone (MP) and methotrexate (MTX) and/or radiotherapy; craniotomy in combination with dexamethasone or MP and/or radiotherapy; and neuroendoscopic surgery in combination with MP and MTX. The follow-up period was 5–27 months with an average duration of 10.1 months. After the initial three months of follow-up, the clinical symptoms of all the patients were significantly improved, with the tumor disappearing in seven patients and evidently reducing in size in two patients. However, six patients exhibited tumor recurrence, three of whom eventually succumbed to the disease during the follow-up period. Currently, comprehensive treatment strategies based on a combination of stereotactic biopsy, chemotherapy and radiotherapy are recommended for the treatment of PCNSL. However, the effectiveness of these treatments remains unsatisfactory. Thus, future studies are required to investigate methods for improving the efficacy of PCNSL treatment strategies.

## Introduction

Primary central nervous system lymphoma (PCNSL) is clinically characterized as a rare and specific type of non-Hodgkin’s lymphoma that is localized in the brain, spinal cord, meninges and eyes, without the involvement of other body systems (1–3). PCNSL exhibits an annual incidence rate of 0.43 per 100,000 individuals in the USA (1,2). The majority of PCNSL patients are male and the median age at diagnosis is ~60 years, with no differences observed between ethnic groups (1–5). It has been reported that PCNSL accounts for 1–3% of all central nervous system (CNS) tumors and 2–3% of non-Hodgkin’s lymphoma cases, while diffuse large B-cell lymphoma accounts for >90% of all PCNSL cases (1–4). Diagnosis of PCNSL is most often obtained using stereotactic biopsy (1,3–8). And the main treatment strategies for PCNSL are chemotherapy and radiotherapy (1–4,6–8). Several novel therapeutic strategies have been developed, including immunotherapy, targeted therapy or stem cell transplantation therapy (4,6–9). PCNSL is a highly aggressive lymphoma with a poor prognosis and a median survival time of nine months following diagnosis (4). Due to its high malignancy and poor prognosis, PCNSL diagnosis and treatment have been a challenge in the neurosurgical field (1,3,4). Therefore, the aim of the present study was to retrospectively analyze the recent clinical data and outcomes of nine PCNSL patients who received treatment at the First Hospital of China Medical University (Shenyang, China) between January 2011 and January 2014. Furthermore, combined with a literature review, the present study reviewed and discussed the possible treatment strategies for this malignant tumor type.

## Case report

The current study included nine patients who were diagnosed with PCNSL at the First Hospital of China Medical University between January 2011 and January 2014. The patients included two males and seven females patients, with ages ranging between 47 and 76 years and an average age of 61.4 years. The present study conformed to the principles outlined in the Declaration of Helsinki (2013) and was approved by the Ethics Committee of China Medical University. Furthermore, written informed consent was obtained from all the patients.

**Figure 1:**
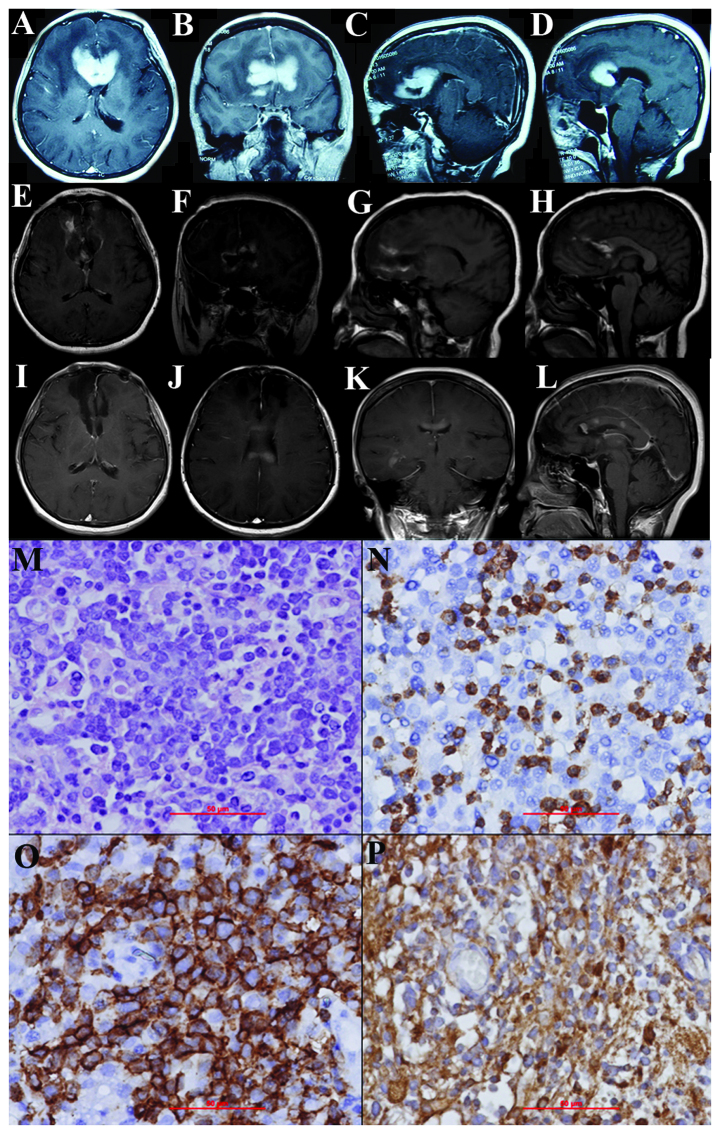
Primary central nervous system lymphoma in the genu of the corpus callosum (case 5). (A-D) Preoperative magnetic resonance imaging (MRI) demonstrated that the lesion displayed a relatively equal-T1 and long-T2 signal on MRI scans. The signals were significantly enhanced using contrast enhancement and a butterfly-shaped pattern was observed. (E-H) Postoperative MRI scans indicating complete removal of the lesion. (I-L) Tumor recurrence with multiple lesions was detected 10 months after surgery in combination with methylprednisolone and methotrexate treatment. Postoperative pathological findings are shown, including (M) hematoxylin and eosin, (N) cluster of differentiation (CD)3-positive, (O) CD20-positive and (P) S-100-positive staining (magnification, ×400; red scale bar = 50 μm).

**Figure 2:**
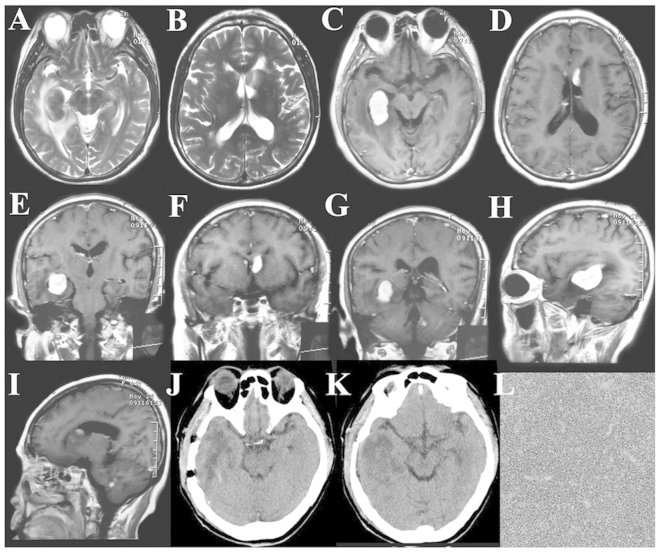
Primary central nervous system lymphoma in multiple regions of the brain (case 8). (A-I) Preoperative magnetic resonance imaging (MRI) scans demonstrated that multiple lesions displayed a relatively equal-T1 and long-T2 signal in the left temporal and occipital lobes, as well as in the right caudate nucleus. The signals were significantly enhanced with the contrast enhancement, demonstrating a fist-like pattern. (J) Intraoperative stereotactic biopsy; (K) postoperative computed tomography on the day of the surgery; (L) postoperative pathological hematoxylin and eosin staining (magnification, ×40).

**Figure 3:**
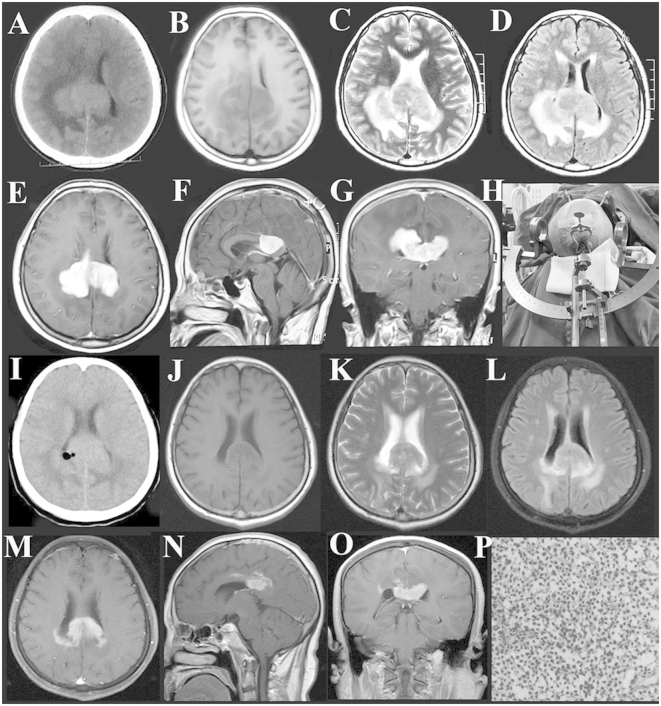
Primary central nervous system lymphoma in the splenium of the corpus callosum (case 9). Preoperative imaging demonstrated that the lesion displayed a high density on (A) computed tomography (CT) scans and an equal-T1 and long-T2 signal on (B-G) magnetic resonance imaging (MRI) scans. The signals were significantly enhanced with the contrast enhancement, demonstrating a butterfly-shaped pattern. (H) Intraoperative stereotactic biopsy; (I) postoperative CT on the day of the surgery. (J-O) MRI scans indicated a marked reduction of the lesion one month after stereotactic biopsy in combination with methylprednisolone, methotrexate and radiotherapy treatment. (P) Postoperative pathological hematoxylin and eosin staining (magnification, ×40).

Preoperative computed tomography (CT) and enhanced magnetic resonance imaging (MRI) scans were performed in the nine enrolled patients, which identified solitary lesions in seven patients and multiple lesions in two patients. Three patients exhibited a solitary lesion in the corpus callosum, one in the left caudate nucleus, one in the left frontal lobe, one in the right cerebellum and one in the anterior cranial fossa and canalis opticus (Figs. 1–3). Furthermore, one patient exhibited multiple lesions in various regions, including the left temporal and occipital lobes, as well as in the right caudate nucleus, whereas another patient exhibited multiple lesions only in the ventricles. All the lesions presented a relatively high intensity on the CT images, while hypertense lesions were observed on the T1-weighted, T2-weighted and fluid-attenuated inversion recovery (FLAIR) images. Following contrast enhancement, distinct, enhanced, mass-like solid structures with typical fist-like, flame-shaped or butterfly-shaped patterns were detected. In addition, significant local edema was identified in the areas surrounding the lesions (Figs. 1–3). Additionally, the disease course of all the patients ranged between 7 days and 10 months, with an average of 3.1 months. The detailed clinical manifestations of each patient are listed in Table I.

The pathological patient specimens were collected in the Department of Neurosurgery of the First Hospital of China Medical University. Following pathological diagnosis, the patients adopted the following chemotherapy and radiotherapy treatment regimens: Two patients underwent craniotomy for tumor removal and were administered with 10 mg/day dexamethasone for six days (craniotomy + dexamethasone); one patient received craniotomy for tumor removal in combination with 10 mg/day dexamethasone for six days and 40 Gy whole-brain radiotherapy (WBRT; craniotomy + dexamethasone + WBRT); two patients received craniotomy in combination with 80 mg methylprednisolone (MP) twice a day (b.i.d) for three days, followed by 40 mg MP b.i.d. for three days, and 3 g/m^2^ methotrexate (MTX; craniotomy + MP + MTX); two patients received stereotactic biopsy in combination with MP and MTX (stereotactic biopsy + MP + MTX); one patient received stereotactic biopsy in combination with MP, MTX and radiotherapy (stereotactic biopsy + MP + MTX + radiotherapy); and one patient received neuroendoscopic surgery in combination with MP and MTX (neuroendoscopic surgery + MP + MTX). Following surgery, all the patients underwent a systemic examination, including whole-body lymph node scanning and chest and abdominal CT scans. In addition, four patients received bone marrow aspiration biopsies and one patient underwent positron emission tomography (PET). The possibility of secondary CNS lymphoma was ruled out as no primary lesions were detected in other body systems of the patients.

Postoperative pathological examination confirmed the diagnosis of non-Hodgkin’s B-cell lymphoma in all the patients, including diffuse large cell lymphoma in four patients, marginal-zone B-cell lymphoma in one patient, activated B-cell subtype in three patients and small B-cell lymphoma in one patient. The detailed pathological and immunochemical results are listed in Table I.

The follow-up duration range was 5–27 months with an average duration of 10.1 months. After the initial three months of follow-up, the clinical symptoms of all the patients were significantly improved. The tumor disappeared in seven patients, while a marked reduction in the tumor size was observed in two patients. However, six patients presented tumor recurrence within the follow-up period; among them, three patients succumbed to the disease, two survived and one was lost to follow-up after 12 months (Table I).

## Discussion

PCNSL is highly aggressive, and the most common histological subtype of PCNSL is diffuse large B-cell lymphoma, with a male to female ratio of 1.2–1.7:1 among PCNSL patients (1,3,5). In the present study, all the PCNSL patients exhibited B-cell lymphoma, accounting for ~0.2% of the total number of patients who were diagnosed with cranial tumors (9/4059 patients) during the time period of the present study in the Department of Neurosurgery of the First Hospital of China Medical University. The incidence rate has exhibited an overall increasing trend in the past 30 years (1,10). This increase is associated with various factors, including the increased number of hospitalized patients, improved diagnostic technologies, increased incidence of HIV infection and administration of immunosuppressive agents following organ transplantation. Of these influencing factors, immunodeficiency is a high risk factor for PCNSL (1,6). For instance, previous studies have identified that the incidence rate of lymphomas is significantly higher in acquired immune deficiency syndrome (AIDS) patients compared with patients not suffering from AIDS (1,10). In particular, the incidence rate of CNS lymphomas reached 5% in AIDS patients prior to the administration of effective antiretroviral therapy, but significantly declined following therapy (1,4).

The clinical manifestations of PCNSL are closely associated with various factors, including the location and number (solitary or multiple) of lesions. Previous studies have revealed that multiple lesions occur in ~34% of PCNSL patients (1,5) and are commonly detected in the frontal lobe. Furthermore, patients with lesions in deep brain tissue, including the corpus callosum, basal ganglia, the area surrounding the ventricles, the brainstem or the cerebellum, accounted for ~40% of PCNSL patients (1,3,6–9). In the present study, seven patients exhibited a solitary lesion and two presented multiple lesions, with the lesion sites being consistent with the aforementioned high-occurrence sites. A recent literature review identified that ~50% of patients exhibited clinical manifestations involving motor and sensory dysfunctions, 30% developed personality changes, 55% experienced headaches, ~35% suffered from nausea, ~10% suffered from vomiting and ~20% of cases were accompanied by uveitis (1). In the present study, five patients experienced headaches, three developed impaired speaking capability, three had limb movement disorders, one suffered from nausea and vomiting, two demonstrated declined memory and responsiveness and one exhibited declined olfactory and visual sensation. These symptoms are consistent with the aforementioned manifestation distribution pattern.

The imaging manifestations of CNS lymphomas demonstrate a relative specificity. The tumor mass predominantly displays a relatively high density (occasionally exhibiting a density similar to the surrounding area) on CT images, with even enhancement observed upon enhanced scanning. In addition, MRI typically demonstrates an equal or low signal on the T1-weighted image and a high signal on the T2-weighted and FLAIR images. Enhanced scanning demonstrates a distinct, even enhancement, with a fist-like, flame-shaped or a unique butterfly-shaped structure in patients with lesions in the corpus callosum (5,6–9). In the present study, the enhanced MRI scans of the patients with lesions in the cerebellum or left frontal lobe and the patients with multiple lesions demonstrated a flame-shaped and fist-like structure, respectively. Furthermore, the enhanced MRI of the three patients with a single lesion in the corpus callosum presented a butterfly-shaped structure. These typical imaging manifestations are conducive to a preliminary diagnosis of a CNS lymphoma. Regarding the differential diagnosis of CNS lymphomas, the common clinical focus is the differential diagnosis from multifocal or multicentric glioma, multiple sclerosis and inflammation. Although specific scan sequences of magnetic resonance spectroscopy, diffusion-weighted images or PET may be used, the authors of the present study consider pathological examination to be the gold standard for the differential diagnosis of CNS lymphomas.

Pathological examination has revealed that the majority of PCNSLs are highly malignant (high grade) and the most common PCNSL type is diffuse large B-cell lymphoma (1,4,8,9). Additionally, light microscope observations have indicated that tumor cells present a diffuse distribution, relatively consistent cell size and small cytoplasmic area. The tumor cell nuclei are medium in size and round or oval in shape, with one or more nucleoli and the presence of mitotic nuclei. The specific immunohistochemical markers of tumor cells are cluster of differentiation (CD)20-positive and CD138-negative (1). In addition, other markers, including multiple myeloma oncogene-1, B-cell lymphoma-6, pan-B-cell antibody, CD19, CD20, CD79α and paired box 5, are typically positive (1). In patients with a clinically normal immune function, the Epstein-Barr virus is predominantly negative, and the mutation frequency of proto-oncogenes, including Pim-1, RhoH/TTF and c-Myc, is two to five-fold higher than in patients with extracranial diffuse large B-cell lymphomas (1). Perivascular large B-cell lymphoma is a pathologically high-malignancy type of PCNSL; however, its occurrence is rare. In addition, low-malignancy (low-grade) PCNSL is rare and predominantly occurs in the spinal cord, with immunocytoma and marginal-zone B-cell lymphoma (mucosa-associated lymphoid tissue type) as two common types. Marginal-zone B-cell lymphomas predominantly occur in female patients (male to female ratio, 1:4) and have an average age of onset of ~49 years and a relatively satisfactory clinical prognosis (1). The present study included one female patient with marginal-zone B-cell lymphoma who did not receive postoperative radiotherapy and succumbed to the disease 10.5 months after surgery.

Primary CNS T-cell lymphoma is rare; thus, the present study did not include any T-cell lymphoma patients. The majority of reported cases are clinically sporadic, accounting for ~2% of PCNSL patients (1,3,4,8,9). The age of onset is ~60 years, and its clinical manifestations and prognosis are similar to B-cell lymphoma (1). CD4-positivity is the major immunohistochemical marker of primary CNS T-cell lymphoma, although other markers may include positive staining for pan T antigens (including CD3 and CD5) (1,4). In addition, a rarer pathological type of PCNSL exists, termed histiocytic sarcoma, which is highly malignant and has poor clinical outcomes (1).

Currently, the diagnostic criteria of PCNSL are relatively definitive, consisting of three major elements: i) The lesions are localized in the brain, spinal cord, meninges and eyes; ii) the lesions in the aforementioned locations are pathologically diagnosed as lymphoma; and iii) the lesions do not involve other parts of the body (1,3–5,8). In the present study, pathological specimens were predominantly collected in the Department of Neurosurgery using stereotactic lesion biopsy, craniotomy for lesion biopsy and cellular pathological examination of cerebrospinal fluid. Stereotactic biopsy is the most common approach and should be the first choice for achieving a definitive pathological diagnosis prior to surgery in patients who are highly suspected to suffer from PCNSL (8).

The effectiveness of PCNSL treatment remains unsatisfactory (1,3,4). Existing studies regarding PCNSL treatment strategies are predominantly retrospective treatment analyses, whereas prospective randomized controlled studies with a large sample size are lacking. Therefore, the optimization of treatment selection requires additional systemic studies to be performed. Currently, the generally accepted treatment strategy for PCNSL is a chemotherapy-based comprehensive treatment (1–4,7–9,11–22). The majority of studies have indicated that the main purpose of the neurosurgical procedure is to obtain a pathological specimen and that no clear association exists between the complete removal of the lesion and patient prognosis (1,7–9,13). However, a previous study identified that the overall survival rate of patients who underwent complete resection or incomplete resection of the tumors was significantly higher compared with patients who received only stereotactic biopsy and particularly in patients with a solitary lesion (14). The majority of researchers consider that chemotherapy (single- or multiple-agent chemotherapy) with or without radiotherapy should be performed at an early stage once the tumor pathological properties are identified (1–4,6–10,12,13,18,19). The most common chemotherapeutic agent used in single-agent chemotherapy is MTX, which is recommended to be administration at a high dose of MTX (2.5–3.5 g/m^2^, up to a maximal dose of 8 g/m^2^) (1,3,10,12,13,16,18). MTX may be administered concurrently with other agents, including vincristine, procarbazine, temozolomide (150 mg/m^2^/day), rituximab (375 mg/m^2^) and dexamethasone (12 mg) (15–17,22), or in combination with whole-brain radiotherapy (WBRT) (1). The response rate to single-agent chemotherapy using MTX is 52–88%, to multiple-agent chemotherapy combining MTX and other chemotherapeutic agents is 70–94%, and to WBRT is ~90% (15). The two-year survival rate of the patients who received single- and multiple-agent MTX chemotherapy combined with WBRT was 58–72% and 43–73%, respectively (15). A previous study has demonstrated that an increase in radiation dose and radiotherapy area did not appear to affect the overall patient survival rate (19). Due to the high radiation dose of up to 45 Gy used in the majority of studies, the neurotoxicity incidence was relatively high (83% within one year among patients aged >60 years), resulting in a marked deterioration in patient cognitive function and quality of life (20). Recent studies identified that patients who underwent high-dose chemotherapy combined with autologous stem cell transplantation exhibited a three-year survival rate of 60% and an event-free survival rate of 53% (16,17). In newly-diagnosed young patients, the outcomes of this treatment strategy were superior (21). In the present study, all the patients were administered hormones following craniotomy or stereotactic biopsy due to a previous finding that lesions in the majority of patients are sensitive to hormones. The underlying mechanism is that hormones induce tumor cell apoptosis and reduce the surrounding edema of the tumor body through binding to glucocorticoid receptors on the surface of tumor cells. A previous study reported that this hormone-induced effect may last for 6–60 months (11); however, the majority of studies have indicated that hormones only temporarily relieve the symptoms (3,4,9,21). Therefore, it was proposed that all the patients in the present study should receive postoperative chemotherapy and radiotherapy. Six patients were selected to undergo subsequent high-dose MTX chemotherapy, while two patients underwent subsequent radiotherapy (regional + whole-brain). The follow-up data indicated a short-term improvement of the clinical manifestations in all the patients, as evidenced completely undetectable lesions in seven patients and significant lesion reduction in two patients. However, six out of seven patients subsequently exhibited tumor recurrence and the recurred tumors progressed rapidly, with three patients succumbing to the disease during the follow-up period. These data indicate a poor prognosis for the PCNSL patients. Currently, the treatment strategies for PCNSL, in particular recurrent PCNSL, remains unsatisfactory.

In conclusion, PCNSL is a rare tumor type in the CNS. Compared with the extracranially-localized large B-cell lymphoma, the effectiveness of the PCNSL treatment is unsatisfactory, which may be due to the lack of sufficient knowledge regarding the biological properties of PCNSL. In addition, the poor permeability of the existing chemotherapeutic agents through the blood-brain barrier may be responsible for the poor prognosis of PCNSL patients. Currently, comprehensive treatment strategies based on a combination of stereotactic biopsy, chemotherapy and radiotherapy are recommended. Developments in targeted biological and gene therapy strategies are expected to improve the treatment efficacy for PCNSL in the future.

## Figures and Tables

**Table I:** Clinical manifestations, treatment regimens, pathological characteristics and prognosis of nine primary central nervous system lymphoma patients.

Case	Gender/Age, years	Clinical manifestation	Lesion site	Treatment regimen	Postoperative pathological diagnosis (immunohistochemical staining)	Follow-up, months/outcome
1	F/68	Headache and dizziness, for one month nausea and vomiting accompanied by unsteady gait for one week	Right brachium pontis	Craniotomy, dexamethasone and radiotherapy	NHBL [CD3 (sporadic+), CD20 (+), CD34 (−), S-100 (−), vimentin (−), CK (−), Ki67 (+>50%), GFAP (−), Pax-5 (+)]	27/Mortality
2	F/58	Deteriorated memory for three months, reduced ability to speak for 10 days	Left frontal lobe	Craniotomy and dexamethasone	NHBL [CD34 (−), pan-CK (−), GFAP (−), Ki-67 (+>75%), S-100 (−), vimentin (−), CD20 (+), CD3 (−), CD38 (low+), CD68 (sporadic+), CD138 (−), IDH1 (−), Pax-5 (++)]	12/Lost
3	F/60	Headache and dizziness for three months	Left caudate nucleus	Craniotomy and dexamethasone	NHBL/marginal-zone B-cell lymphoma [CK (−), vimentin (−), GFAP (+), S-100 (−), synaptophysin (−), CD3 (low+), CD20 (−), Pax-5 (+), Ki67 (+>30%), CD138 (−), κ (+), λ (−), CD5 (low+), CD23 (−), cyclin D1 (−)]	9.8/Mortality
4	M/47	Headache for 10 months; headache became worse and was accompanied by difficult movement of the left extremity for two weeks	Splenium of the corpus callosum	Stereotactic biopsy, MP, MTX and radiotherapy	Diffuse large B-cell lymphoma, germinal center subtype [CD20 (low+), CD21 (+), CD3 (sporadic+), CD56 (−), pan-CK (−), GFAP (+), Ki-67 (+75%), P53 (−), P63 (sporadic+), Pax-5 (+), S-100 (−), synaptophysin (−), TTF-1 (−), vimentin (±), Bcl-6 (sporadic+), CD10 (+), MUM1 (+)]	10/Mortality
5	F/49	Headache and dizziness, deteriorated memory, slow response for six months	Genu of corpus callosum	Craniotomy, MP and MTX	Diffuse large B-cell lymphoma [CK (−), vimentin (+), GFAP (+), S-100 (local+), synaptophysin (local+), P53 (++), CD3 (sporadic+), CD20 (+), Pax-5 (+), Ki67 (+50%), Bcl-6 (+), CD10 (−), MUM1 (+)]	13/Remission
6	F/76	Declined olfactory sensation for one month and visual decline for two weeks	Floor of the anterior cranial fossa, canalis opticus	Neuroendoscopic removal of the majority of the tumor, MP and MTX	Diffuse large B-cell lymphoma, activated B-cell subtype [CK (+), CD3 (−), CD20 (−), Pax-5 (+), CD30 (−), Ki-67 (+>50%) CD56 (−), CD99 (−), synaptophysin (−), Bcl-6 (−), CD10 (−), MUM1 (+), GFAP (−)]	8/Remission
7	M/69	Intermittent headaches and dizziness for three months, unclear speech for three days	Left temporal and occipital lobe, right caudate nucleus	Stereotactic biopsy, MP and MTX	Diffuse large B-cell lymphoma, activated B-cell subtype [Bcl-6 (−), CD10 (−), CD20 (++), CD3 (+), CD30 (−), CK (−), EMA (−), GFAP (−), Ki-67(80%), MUM1 (+), Pax-5 (+), S-100 (−), synaptophysin (−), TTF-1 (−), vimentin (+), CD79a (±)]	6
8	F/65	Headache and dizziness for 10 days	Temporal and frontal horn of lateral ventricle, fourth ventricle	Craniotomy, MP, dexamethasone and MTX	NHBL, activated B-cell subtype [CD3 (+), CD20 (+), Pax-5 (+), CD10 (−), Bcl-6 (−), MUM1 (+), CD30 (−), Ki-67 (+>80%), CD15 (−), CD68 (+)]	5
9	F/61	Difficult movement of the left extremity for one week	Splenium of the corpus callosum	Stereotactic biopsy, dexamethasone and MTX	NHBL [CD20 (+), CD3 (sporadic+), pan-CK (−), GFAP (−), Ki-67 (+80%), NSE (+), S-100 (−), synaptophysin (−), vimentin (+), AFP (+), hCG (−), PLAP (−), Pax-5 (+), Bcl-6 (+), CD10 (−), MUM1 (+)]	5

F, female; NHBL, non-Hodgkin’s B-cell lymphoma; CD, cluster of differentiation; CK, cytokeratin; GFAP, glial fibrillary acidic protein; Pax-5, paired box-5; M, male; IDH1, isocitrate dehydrogenase 1; MP, methylprednisolone; MTX, methotrexate; TTF-1, thyroid transcription factor 1; Bcl, B-cell lymphoma; MUM1, multiple myeloma oncogene 1; EMA, epithelial membrane antigen; NSE, neuron-specific enolase; AFP, α-fetoprotein; hCG, human chorionic gonadotropin; PLAP, phospholipase A2-activating protein.
